# Association between Gut Microbiota Composition and Long-Term Vaccine Immunogenicity following Three Doses of CoronaVac

**DOI:** 10.3390/vaccines12040365

**Published:** 2024-03-27

**Authors:** Li-Na Zhang, Jing-Tong Tan, Ho-Yu Ng, Yun-Shi Liao, Rui-Qi Zhang, Kwok-Hung Chan, Ivan Fan-Ngai Hung, Tommy Tsan-Yuk Lam, Ka-Shing Cheung

**Affiliations:** 1 Department of Medicine, School of Clinical Medicine, The University of Hong Kong, Queen Mary Hospital, Hong Kong; 2 School of Clinical Medicine, The University of Hong Kong, Hong Kong; 3 State Key Laboratory of Emerging Infectious Diseases, School of Public Health, The University of Hong Kong, Hong Kong; 4 Centre for Immunology & Infection Limited, 17W Hong Kong Science & Technology Parks, Hong Kong; 5 Department of Microbiology, School of Clinical Medicine, The University of Hong Kong, Queen Mary Hospital, Hong Kong

**Keywords:** gut microbiota, COVID-19 vaccine, vaccine immunogenicity

## Abstract

Background: Neutralizing antibody level wanes with time after COVID-19 vaccination. We aimed to study the relationship between baseline gut microbiota and immunogenicity after three doses of CoronaVac. Methods: This was a prospective cohort study recruiting three-dose CoronaVac recipients from two centers in Hong Kong. Blood samples were collected at baseline and one year post-first dose for virus microneutralization (vMN) assays to determine neutralization titers. The primary outcome was high immune response (defined as with vMN titer ≥ 40). Shotgun DNA metagenomic sequencing of baseline fecal samples identified potential bacterial species and metabolic pathways using Linear Discriminant Analysis Effect Size (LEfSe) analysis. Univariate and multivariable logistic regression models were used to identify high response predictors. Results: In total, 36 subjects were recruited (median age: 52.7 years [IQR: 47.9–56.4]; male: 14 [38.9%]), and 18 had low immune response at one year post-first dose vaccination. *Eubacterium rectale* (log_10_LDA score = 4.15, *p* = 0.001; relative abundance of 1.4% vs. 0, *p* = 0.002), *Collinsella aerofaciens* (log_10_LDA score = 3.31, *p* = 0.037; 0.39% vs. 0.18%, *p* = 0.038), and *Streptococcus salivarius* (log_10_LDA score = 2.79, *p* = 0.021; 0.05% vs. 0.02%, *p* = 0.022) were enriched in low responders. The aOR of high immune response with *E. rectale, C. aerofaciens*, and *S. salivarius* was 0.03 (95% CI: 9.56 × 10^−4^–0.32), 0.03 (95% CI: 4.47 × 10^−4^–0.59), and 10.19 (95% CI: 0.81–323.88), respectively. *S. salivarius* had a positive correlation with pathways enriched in high responders like incomplete reductive TCA cycle (log_10_LDA score = 2.23). *C. aerofaciens* similarly correlated with amino acid biosynthesis-related pathways. These pathways all showed anti-inflammation functions. Conclusion: *E. rectale,* *C. aerofaciens*, and *S. salivarius* correlated with poorer long-term immunogenicity following three doses of CoronaVac.

## 1. Introduction

As of December 2023, worldwide Coronavirus disease 2019 (COVID-19) infections have surpassed 700 million, resulting in over six million deaths [1]. Efficient vaccines and effective vaccination programs were among the most useful solutions in combating the pandemic. However, it has been reported that antibody levels naturally wane over time, with an estimated halving every 108 days [2]. One study had demonstrated that the effectiveness of the CoronaVac vaccine against COVID-19 infection decreased from 74.5% to 30.4% after 3–5 months [3]. A steady decline in antibody titers was also observed in the Hong Kong cohort six months after receiving the second dose of either the CoronaVac (inactivated virus) or BNT162b2 (mRNA) vaccine [4]. 

An increasing number of studies suggested that microbiota could modulate the hosts’ baseline immune status and influence their immune response [5]. It was reported that certain bacterial species facilitated the activation of antigen-presenting cells, such as dendritic cells (DCs), through pattern recognition receptors (PRRs) or nucleotide-binding oligomerization domain-like receptors (NLRs) [6]. This hypothesis was supported by findings related to Toll-like receptor 5 (TLR5)-mediated sensing of flagellin and the requirement for peptidoglycan by Nucleotide-binding Oligomerization Domain2 (NOD2) in intranasal immunization [7,8]. Moreover, bacteria related to *Clostridia* were also found contributing to the development of T helper 17 cells and Forkhead box P3 regulatory T (FOXP3+ Treg) cells, besides stimulating the production of pro-inflammatory cytokines [9,10]. The influence of microbiota on immunoglobulin A (IgA) secretion of B cells and differentiation of lymphoid cells was also reported in prior studies [11,12]. Another theory suggests that certain metabolites derived from microbiota, such as short-chain fatty acids (SCFAs) [13], secondary bile acids [14], lipopolysaccharides (LPS) [15], flagellin [16], and peptidoglycans [17], play a role in regulating response to vaccines. A recent study has demonstrated that microbiota might encode cross-reactive antigens to pre-activate correlated immune responses and enhanced subsequent responses to vaccines. However, further validation is required to confirm this theory [18]. The association between microbiota and the immune response was further confirmed by observations from a randomized controlled trial, which showed that antibiotic-induced gut microbiota dysbiosis reduced antibody levels following influenza vaccination [19].

Accumulating evidence suggests the potential correlation between microbiota and the immune response to COVID-19 vaccination [20]. A study involving subjects with two doses of vaccination found that *Bifidobacterium adolescentis* was positively correlated with a higher immune response to the CoronaVac after one month, while bacteria like *Roseburia faecis*, featuring flagella and fimbriae, were linked to a stronger immune response to the BNT162b2 vaccine within the same timeframe [21]. Another study, examining a population with inflammatory bowel disease (IBD) over a 100-day dynamic follow-up, found that higher *Bilophila* levels were associated with improved serological responses, while elevated *Streptococcus* levels were correlated with weaker responses. Additionally, the study observed that metabolites such as trimethylamine, isobutyrate, and omega-muricholic acid were linked to better responses, whereas succinate, phenylalanine, taurolithocholate, and taurodeoxycholate were associated with poorer responses [22]. 

In view of the waning vaccine immunogenicity, a booster dose has been recommended to sustain the protective level of antibodies. Nevertheless, its decline remains inevitable [23,24]. The existing evidence on gut microbiota and vaccine immunogenicity is only limited to two doses of vaccines [25,26]. To fill in the knowledge gap, our study aimed to investigate the association between baseline microbiota and long-term immune response at one year in recipients that were administered three doses of CoronaVac.

## 2. Methods

### 2.1. Study Cohort

Adult subjects who had completed three doses of CoronaVac [27] (Sinovac Life Sciences Co., Ltd., Beijing, China) at two vaccination centers in Hong Kong (Sai Ying Pun Jockey Club Clinic and Tung Wah Hospital Staff Clinic) were enrolled in this prospective cohort study. Subjects received first two doses of intramuscular CoronaVac (0.5 mL/per dose) 28 days apart. The booster was administered at least 6 months after the first two doses in non-immunocompromised adults aged 18 years or above. 

Exclusion criteria included subjects with (i) age below 18; (ii) inflammatory bowel disease (IBD), immunocompromised state (including post-transplant [28] and on immunosuppressives/chemotherapy), and conditions like cancer, hematological, rheumatological and autoimmune diseases; and (iii) COVID-19 over the course of study or prior infection. Subjects with a history of COVID-19 or presence of severe acute respiratory syndrome coronavirus 2 (SARS-CoV-2) nucleocapsid protein antibodies, which are not induced by current vaccines, were identified as having past infection. This study was approved by the Institutional Review Board (IRB) of the University of Hong Kong and the Hospital Authority Hong Kong West Cluster (UW 21-216). Informed consent forms were signed by all subjects. 

### 2.2. Collection of Demographics as Well as Blood and Stool Samples

Data such as demographics (age, sex), anthropometrics (body mass index (BMI)), lifestyle (smoking and drinking habits), medical history (diabetes (DM) or pre-DM, metabolic dysfunction-associated steatotic liver disease (MASLD) [29,30], hypertension, surgical history, medication history (proton pump inhibitors, antibiotics [31,32] within a year before vaccination), and liver stiffness measurement [28] were collected. OWOB was defined as a BMI of ≥23 kg/m^2^. Pre-DM was defined as fasting plasma glucose level of 5.6–6.9 mmol/L or glycated hemoglobin level of 5.7–6.4% according to criteria proposed by the American Diabetes Association [33]. Liver stiffness was expressed in terms of fibroscore obtained with transient elastography (Fibroscan, Echosens^®^, Paris, France) [34]. MASLD was identified according to the American Association for the Study of Liver Diseases (AASLD) guideline [35]. Blood samples for virus microneutralization assay were collected (i) before vaccination (baseline) and (ii) one year after the first dose.

In our study, vaccine immunogenicity was evaluated using the virus microneutralization (vMN) assay. COVID-19 and SARS-CoV-2 vaccines can induce the production of non-neutralizing and neutralizing antibodies, both of which can bind to the virus. However, only neutralizing antibodies can prevent the host from virus infection. Various methods have been utilized to assess the antibody level, including enzyme-linked immunoassay (ELISA), immunofluorescence (IF), and vMN. ELISA and IF can detect antibody binding to viral antigens or the virus, while vMN titers specifically reveal the neutralizing activity against the virus. Both the receptor-binding domain (RBD) and the N-terminal domain (NTD) of the spike protein can induce anti-SARS-CoV-2 neutralizing antibodies, which can inhibit virus infection by blocking viral entry [36]. Currently, there is an ELISA-based surrogate neutralizing antibody (sNAb) test, which can be used to evaluate anti-RBD antibodies [37]. However, vMN results represent the total neutralizing activity, including both anti-RBD and anti-NTD neutralizing antibodies, thereby indicating the overall level of protection. Viral neutralization tests (VNTs) are commonly acknowledged as the ‘gold standard’ for serological detection due to their ability to demonstrate the inactivation of infectious virus [38]. Consequently, VNTs serve as a robust correlative measure indicative of immunity and protection against disease. In the vMN assay, serum samples were serially diluted 2-fold in minimum essential medium (Gibco, Green Island, NY, USA), starting at a 1:10 dilution. These diluted sera were mixed with 100 microliters of a 50% tissue culture infective dose (TCID50) of SARS-CoV-2 virus HKU-001a (wild type; Genbank accession number MT230904) and incubated at 37 °C for 1.5 h. The mixture was then added to VeroE6 TM-PRSS2 cells and incubated at 37 °C with 5% CO_2_ for 72 h. The cytopathic effect (CPE) was observed under an inverted microscope after co-incubation. 

The vMN titer was defined as the highest serum dilution at which the CPE percentage equaled 50%, adhering to the World Health Organization’s International Standard for measurements of human antibodies against SARS-CoV-2. The pooled plasma (NIBSC code 20/136) utilized in the vMN assay was sourced from 11 patients that recovered from COVID-19, with each ampoule standardized to 250 IU (https://www.nibsc.org/documents/ifu/20-136.pdf, accessed on 19 February 2024). A vMN titer of 10 (31.25 IU/mL) or greater was considered as seroconversion (seropositivity), while samples lacking a qualified CPE percentage at the starting dilution (1:10) were classified as non-seroconversion (seronegativity) and labelled as “undetectable”. Live virus experiments were conducted in a biosafety level 3 facility.

### 2.3. Primary Outcome of Interest

The primary outcome of interest was persistent high immune response at one year. We defined the top 50% of the cohort with vMN titer greater than or equal to 1:40 at one year as high immune responders. The seroconversion was not used for primary outcome as 97.2% of the cohort had vMN titer greater than or equal to 1:10 at one-year timepoint.

### 2.4. Shotgun Metagenomic Sequencing of Stool Samples

Stool samples were collected by patients and transported to laboratory within 48 h. Baseline fecal samples were collected before the first vaccination, preserved in the OMNIgene tube, and stored at −80 °C [39] (further details can be found in Supplementary File). The total genomic DNA was obtained with Qiagen QIAamp DNA stool Mini Kit (Qiagen, Hilden, Germany). In short, the fecal pellet was suspended in 1 mL of InhibitEX Buffer, vortexed, and centrifuged to disperse the fecal particles. Then, 600 μL of the supernatant was mixed with 25 μL of Proteinase K and 600 μL of Buffer AL, and the mixture was heated at 70 °C for 10 min. Subsequently, 600 μL of ethanol (96–100%) was added to the lysate and filtered through centrifugation. Following this, 500 μL of Buffer AW2 was used to wash the yield. Finally, 200 μL of Buffer ATE was utilized to elute and collect the extracted DNA. The primary DNA was then subjected to library construction using Nextera DNA Library Prep Kit (Illumina, CA, USA). Briefly, the genomic DNA was initially fragmented and labeled with adapter sequences using engineered transposomes, followed by limited-cycle polymerase chain reaction (PCR) to attach the index adapter sequences. Subsequently, the PCR products were purified using AMPure XP beads (Beckman-Coulter, CA, USA). Post-preparation, the library was assessed by a Qubit fluorometer (Thermo Fisher Scientific, MA, USA) and a Bioanalyzer (Agilent Technologies, CA, USA). Upon confirming the library’s quality, high-throughput shotgun metagenomic sequencing was performed on the Illumina NovaSeq 6000 platform (150 bp pairs paired-end), generating 10 Gb raw data per sample.

### 2.5. Bioinformatics Analysis

Raw data were filtered and trimmed by fastp v0.20.1 [40] to remove the adapters and bases with poor quality, and subsequently processed by Bowtie2 [41] to remove host sequence through mapping reads against human reference genome GRCh38.p13. The composition of microbial species and functional profile were identified with the refined reads using MetaPhlAn (v3.0) [42] and HUMAnN (v3.0) [43], respectively. Estimation of species coverage and relative abundance was determined. R statistical software (R Foundation for Statistical Computing, Vienna, Austria, version 4.3.2) was used for all statistical analyses. The putative bacterial species and metabolic pathways were identified using linear discriminant analysis effect size (LEfSe, version 1.1.2) analysis. Species and pathways with a linear discriminant analysis (LDA) score exceeding 2 and a *p* value below 0.05 were considered as differentially enriched. To reduce the risk of false positives, only bacterial species with a median relative abundance greater than 0 in both groups were included in the subsequent analysis.

### 2.6. Statistical Methods

Data were presented as the median (interquartile range [IQR]) for continuous variables, and as the number of subjects (percentage) for categorical variables. The Mann–Whitney U test was used for the assessment of two continuous variables, and the Fisher’s exact test was used for categorical variables between the two groups, respectively. Spearman’s correlation tests were employed to assess the correlations between bacterial species and metabolic pathways. The false discovery rate (FDR) was employed to adjust for multiple comparisons during multiple hypothesis testing [44].

Multivariable logistic regression model was used to calculate the adjusted OR (aOR) and *p*-value, respectively, of high immune response with different clinical factors and high abundance of putative bacterial species identified through LEfSe analysis. We defined the 25% population (i.e., above 75 percentile) of the bacterial species as high abundance. Generalized linear model (GLM) was performed to construct predictive models using bacterial species, and the Receiver Operating Characteristic (ROC) curve was used to show the predictive value. The area under the ROC curve (AUROC) was then utilized to evaluate the accuracy of the model. A two-sided *p*-value of <0.05 was considered statistically significant. 

## 3. Results

### 3.1. Baseline Characteristics 

A total of 36 recipients that were administered three doses of CoronaVac were enrolled, of which 61.1% (*n* = 22) were female (Figure S1). The median age of the participants was 52.7 years (IQR: 47.9–56.4). Among the subjects, 23 (63.9%) were OWOB, 17 (47.2%) had DM or pre-DM, and 13 (36.1%) had MASLD. Eighteen (50%) recipients with a one-year vMN titer of 20 or less (range: undetectable–20) were categorized as the low immune response group, while the other eighteen with vMN above 20 (range: 40–640) were classified as the high immune response group. Baseline characteristics were comparable between the two groups (Table S1). 

### 3.2. Baseline Gut Microbiota Was Associated with Three-Dose CoronaVac Immunogenicity at One Year

**Figure 1:**
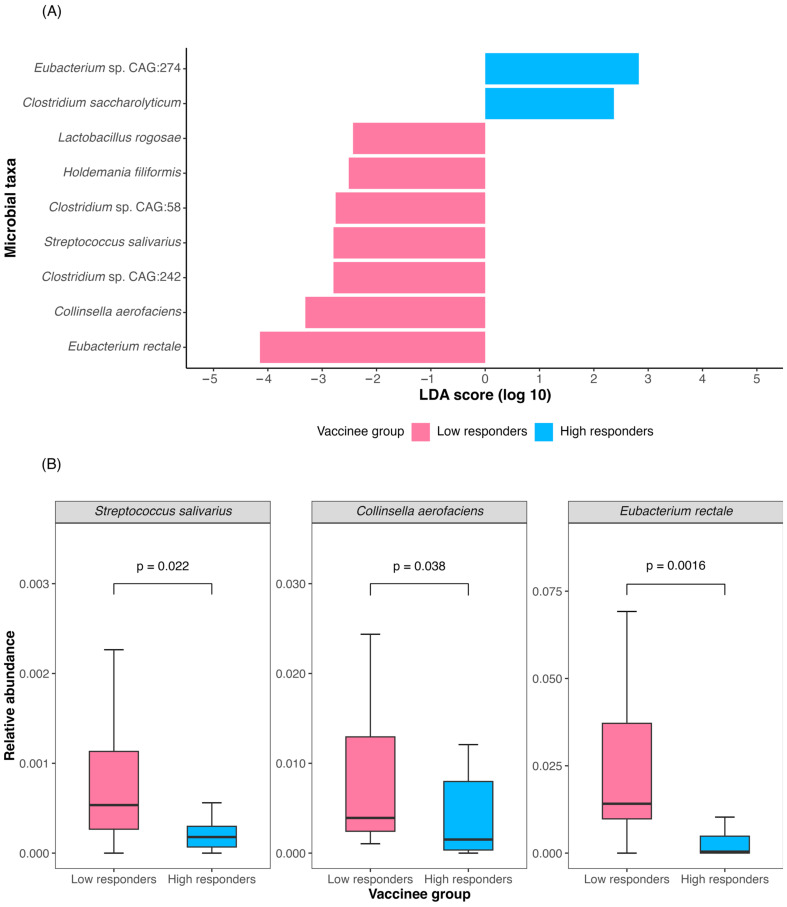
**Baseline bacterial species enrichment and relative abundance in low and high immune response group one year after CoronaVac vaccination.** (**A**) Bacterial species differentially enriched between the low and high response groups based on LEfSe analysis. (**B**) Relative abundance of species differentially enriched with non-zero abundance in both low and high response groups.

In the low response group, seven species were enriched using LEfSe analysis (Figure 1A). Among them, three species were not zero-inflated: *Eubacterium rectale* (log_10_LDA score = 4.15, *p* = 0.001; relative abundance of 1.41% vs. 0, *p* = 0.002), *Collinsella aerofaciens* (log_10_LDA score= 3.31, *p* = 0.037; relative abundance of 0.39% vs. 0.18%, *p* = 0.038), and *Streptococcus salivarius* (log_10_LDA score= 2.79, *p* = 0.021; relative abundance of 0.05% vs. 0.02%, *p* = 0.022) (Figure 1A,B). Although two bacterial species, *Eubacterium sp CAG 274* and *Clostridium saccharolyticum*, were found to be abundant in high response group using LEfSe analysis, their abundance was zero-inflated, indicating that their median abundance was absent in one of the groups.

On multivariable analysis, the OR of high immune response with high abundance of *Eubacterium rectale, Collinsella aerofaciens*, and *Streptococcus salivarius* was 0.03 (95% CI: 9.56 × 10^−4^–0.32), 0.03 (95% CI: 4.47 × 10^−4^–0.59), and 10.19 (95% CI: 0.81–323.88), respectively (Table 1). 

The predictive power of *Eubacterium rectale* for high immune response was higher than that of *Collinsella aerofaciens* (AUROC: 0.82 vs. 0.68) and not different from the model combining these two bacterial species (AUROC: 0.82 vs. 0.83; Figure S2).

### 3.3. Baseline Metabolic Pathways Were Associated with Three-Dose CoronaVac Immunogenicity at One Year

**Figure 2:**
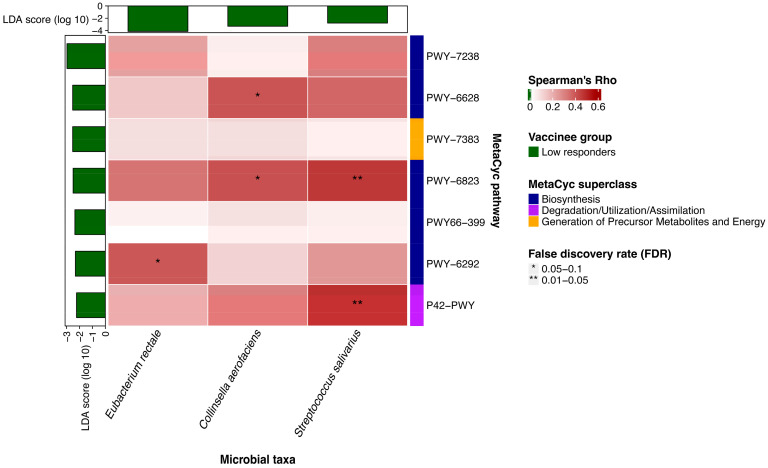
**Correlations between baseline microbiota species and gut metabolic pathways.** Heatmap illustrating the correlations between the relative abundance of microbiota species and metabolic pathways, analyzed using Spearman’s correlation analysis.

Seven metabolic pathways were enriched in the low response group, as shown in Figure S3. These pathways were categorized into three major superclasses according to the MetaCyc database: ‘Biosynthesis’, ‘Generation of Precursor Metabolites and Energy’, and ‘Degradation/Utilization/Assimilation’ (Table S2). Most of these pathways were related to biosynthetic processes like the superpathway of L-cysteine biosynthesis (mammalian) (log_10_LDA score = 2.32, *p* = 0.029) and the superpathway of L-phenylalanine biosynthesis (log_10_LDA score = 2.53, *p* = 0.016). There were also pathways involved in energy generation procedures, including incomplete reductive TCA cycle (log_10_LDA score = 2.23, *p* = 0.018) and anaerobic energy metabolism (invertebrates, cytosol) (log_10_LDA score = 2.53, *p* = 0.018). 

(Figure 2 and Figure S4, Table S3) demonstrate the correlation between metabolic pathways, bacteria species, and the vMN titers evaluated using Spearman’s correlation analysis. The enrichment of the superpathway of L-phenylalanine biosynthesis (r = 0.40; *p* = 0.015) and molybdopterin biosynthesis (r = 0.41; *p* = 0.013) was positively correlated with the abundance of *Collinsella aerofaciens.* A positive association was also observed between the superpathway of L-cysteine biosynthesis and *Eubacterium rectale* (r = 0.40; *p* = 0.017), as well as between molybdopterin biosynthesis and incomplete reductive TCA cycle and *Streptococcus salivarius* (r = 0.46; *p* = 0.004 and r = 0.48; *p* = 0.003, respectively). Additionally, all seven identified metabolic pathways were negatively correlated with vMN titers, and six of these correlations were statistically significant.

## 4. Discussion

Our study is the first to explore the association between the baseline gut microbiota composition and the immune response following three doses of CoronaVac with a comprehensive one-year follow-up. We identified that a high abundance of *Eubacterium rectale*, *Collinsella aerofaciens*, and *Streptococcus salivarius* is is associated with a poor immune response post-vaccination. The association is most obvious in *Eubacterium rectale* species. We also identified seven metabolic pathways enriched in seronegative individuals that are linked to physiological activities such as amino acid biosynthesis and energy metabolism. Notably, most of these pathways were positively correlated with *Streptococcus salivarius* and *Collinsella aerofaciens*.

*Streptococcus salivarius* was demonstrated to have anti-inflammatory effects on hosts by inhibiting the activation of the NF-κB pathway on intestinal epithelial cells and increasing the level of anti-inflammatory cytokine IL-10 on human peripheral blood mononuclear cells (PBMCs) [45]. This pathway was also verified on colitis mouse models [45,46]. NF-κB signaling plays a key role in the immune reaction to vaccines by producing pro-inflammatory cytokines like IL-6 and IL-8, and mediating the maturation and activation of various immune cells [47,48,49]. *Collinsella aerofaciens* was reported to be abundant in stool samples with high SARS-CoV-2 infectivity [50], suggesting a potential correlation between this species and poor immune response and disease recovery. *Collinsella aerofaciens* and *Eubacterium rectale* are butyrate-producing bacteria [51,52]. Butyrate, a member of the SCFAs, is widely recognized as a positive factor in immunity homeostasis and exhibits anti-inflammatory functions to prevent overreactions to infections [51,53]. Butyrate can interact with G protein-coupled receptors GPR41 and GPR43 and histone deacetylase (HDAC) on Treg cells to promote IL-10 secretion and alleviate the activation of NF-κB signaling [54,55]. This interaction may explain why a high abundance of these two species could lead to a low immune response. 

We observed that the abundance of *Eubacterium rectale* alone can effectively distinguish between individuals with high and low immune responses, achieving a satisfactory AUROC at 0.82, which is not significantly different from the combined models. This indicates that the baseline abundance of *Eubacterium rectale* may serve as a reliable predictor of an immune response after vaccination. Identifying individuals with low immune responses during baseline examinations opens the possibility for targeted interventions, enhancing protection for recipients and minimizing overall infectious risks. 

Seven metabolic pathways were enriched in the low response group. The incomplete reductive TCA cycle utilizes acetyl-CoA derived from acetate, and its upregulation often leads to a consumption of acetate in the gut [56,57,58]. Acetate can amplify Toll-like receptor 2 (TLR2) signaling in CD4 T cells and thereby promote the IgA production against certain microorganisms, which has also been verified by animal models [12,59]. Furthermore, an elevated level of acetate can enhance the activities of glyceraldehyde-3-phosphate dehydrogenase (GAPDH), thereby augmenting the glycolytic reserve of rapid memory CD8+ T cells and boosting their function [60]. Therefore, a reduced level of acetate in the gut may lead to a weakened immune response during infections. Moreover, the incomplete reductive TCA cycle pathway omits steps involved in synthesizing citrate found in normal TCA cycles [61,62]. However, the iron salt ferric ammonium citrate (FAC) was reported to block infections by Influenza A virus, ZIKA virus and HIV, and contribute to recovery [63]. In vivo experiments showed that FAC significantly inhibits viral replication and improves survival rates. Therefore, upregulation of the incomplete reductive TCA cycle may lead to a lower immune response. The enrichment in the anaerobic energy metabolism pathway (invertebrates, cytosol) may further enhance the incomplete reductive TCA cycle through anaerobic metabolism [64]. This pathway also leads to lactate accumulation, creating an acidic environment that suppresses a host’s immunity [65]. Meanwhile, increased lactate has been reported to produce a stop-migration signal in T cell effectors under inflammatory conditions and inhibit the synthesis of TNF-α [66,67].

The superpathway of L-cysteine biosynthesis (mammalian) can produce L-cysteine to downregulate the production of pro-inflammatory cytokines like IL-6, TNF-α, interferon-γ (IFN-γ), and IL-1β in inflammatory bowel disease (IBD) [68]. L-cysteine can be utilized to synthesize N-acetyl-cysteine (NAC) [69], which inhibits the basal NF-κB activity in DCs and simultaneously downregulates the expression of surface molecules known to be critical for their antigen-presenting cell (APC) function [70]. Additionally, NAC suppresses T cell proliferation and the synthesis of cytokines of both Th1-type (such as IFN-γ) and Th2-type (such as IL-5) [71]. The serum level of L-phenylalanine is found upregulated in patients with COVID-19 compared to healthy controls [72]. Metabolism of the L-phenylalanine can lead to a rise in hydrogen peroxide (H_2_O_2_), which later suppresses T cell proliferation and function [73,74]. 

A positive correlation was found between the enrichment of the superpathway of L-phenylalanine biosynthesis, the molybdopterin biosynthesis pathway, and the abundance of *Collinsella aerofaciens*. Similarly, a correlation exists between the enrichment of the superpathway of L-cysteine biosynthesis in mammals and the abundance of *Eubacterium rectale*, as well as between the enrichment of the molybdopterin biosynthesis pathway, the incomplete reductive TCA cycle pathway, and the abundance of *Streptococcus salivarius*. These findings may suggest a potential mechanism by which these bacterial species influence the immune response through metabolic processes.

However, our study has several limitations. First, our sample size is small. Second, our research did not investigate the underlying molecular mechanisms by correlating metabolites with bacterial species on vaccine immunogenicity. The correlation between metabolite abundance and microbiota composition may validate the hypothesis that bacteria modulate immunogenicity by altering the production of specific metabolites. Consequently, modulating levels of certain metabolites, such as short-chain fatty acids (SCFAs) and secondary bile acids, could be considered a strategy for enhancing immunogenicity. Moreover, longitudinal analyses, anti-inflammatory mediator measurements, and more follow-up intervals and information about other vaccinations will be essential for future research. 

## 5. Conclusions

*Eubacterium rectale*, *Collinsella aerofaciens*, and *Streptococcus salivarius* were correlated with a poorer immune response at one year following three doses of CoronaVac. These findings may lay the foundation for future research to offer a novel approach by leveraging gut microbiota to enhance the long-term durability of CoronaVac immunogenicity.

## Figures and Tables

**Table 1:** Multivariable logistic regression analysis of factors associated with high immune response.

	aOR	95% CI	*p*-Value
Age ≥ 60 years	0.64	0.04–7.91	0.725
Male sex	3.72	0.40–65.06	0.290
BMI	1.80	1.07–4.16	0.080
DM or pre-DM	26.16	1.61–3.27 × 10^3^	0.071
MASLD	0.20	0.01–2.50	0.229
Proton pump inhibitor use *	0.93	7.29 × 10^−3^–53.12	0.973
Antibiotic use *	146.11	1.23–1.07 × 10^5^	0.068
Fibrosis score	0.42	0.12–1.03	0.106
*Eubacterium rectale ^#^*	0.03	9.56 × 10^−4^–0.32	0.015
*Collinsella aerofaciens ^#^*	0.03	4.47 × 10^−4^–0.59	0.042
*Streptococcus salivarius ^#^*	10.19	0.81–323.88	0.111

* Usage with 1 year before first vaccination. ^#^ High abundance was defined as the top 25% (i.e., above 75 percentile). Abbreviations: OR, odds ratio; 95% CI, 95% confidence interval; aOR, adjusted odds ratio; BMI, body mass index; DM or pre-DM, diabetes mellitus or pre-diabetes mellitus, respectively; MASLD, metabolic dysfunction-associated steatotic liver disease.

## Data Availability

The data presented in this study are available upon request from the corresponding author due to confidentiality issues.

## Supplementary Materials

The following supporting information can be downloaded at: https://www.mdpi.com/article/10.3390/vaccines12040365/s1, Figure S1: Patient recruitment flow diagram; Figure S2. Receiver Operating Characteristic (ROC) curve and Area Under the ROC Curve (AUROC) with 95% confidence intervals for predictive models; Figure S3. Differential enrichment of gut metabolic pathways in low and high immune response group based on LEfSe analysis; Figure S4. Correlation between metabolic pathways and virus microneutralization (vMN) titers; Table S1. Baseline characteristics’ comparison between subjects with low and high immune response at one-year post-vaccination; Table S2. Summary of the identified metabolic pathways; Table S3. Correlation between metabolic pathways and virus microneutralization (vMN) titers based on Spearman’s correlation analysis.
