# Supplementary material for: Association between Gut Microbiota Composition and Long-Term Vaccine Immunogenicity following Three Doses of CoronaVac
Source: Vaccines (Basel). 2024 Mar 27;12(4):365. doi: 10.3390/vaccines12040365 (PMC11055114; doi:10.3390/vaccines12040365)
Supplement: Supplementary file 1 [file vaccines-12-00365-s001.zip › vaccines-2888333-supplementary.pdf]

Stool sample collection and storage[1]

These stool samples were self-collected by patients at home following the manufacturer's instruction (OMNIgene•GUT | OM-200, DNA Genotek Inc., Ottawa, Canada). There are five steps: i). Unscrew the purple cap of the tube. ii). Use the attached spatula to get a small amount of fresh stool sample. iii). Put the stool sample into the yellow tube and scape to level the top of the sample. iv). Screw the purple cap tightly onto the yellow tube top and shake the tube vertically for at least 30 seconds as fast and hard as possible. v). Observe the sample mixed well with the liquid. Only small number of particles left suspended.

The collected samples were brought to us by patients within 48 hours. We then transferred the liquid from the OMNigene tube to an Eppendorf tube and stored it at -80°C upon it was received.

Reference

(1) *DNA Genotek - Support - Collection Instructions - DNA - RNA - Infectious Disease - Animal Genetics*. https://dnagenotek.com/ROW/support/collection-instructions/omnigene-gut/OM-200.html (accessed on 19 February 2024).

Figure S1. Patient recruitment flow diagram

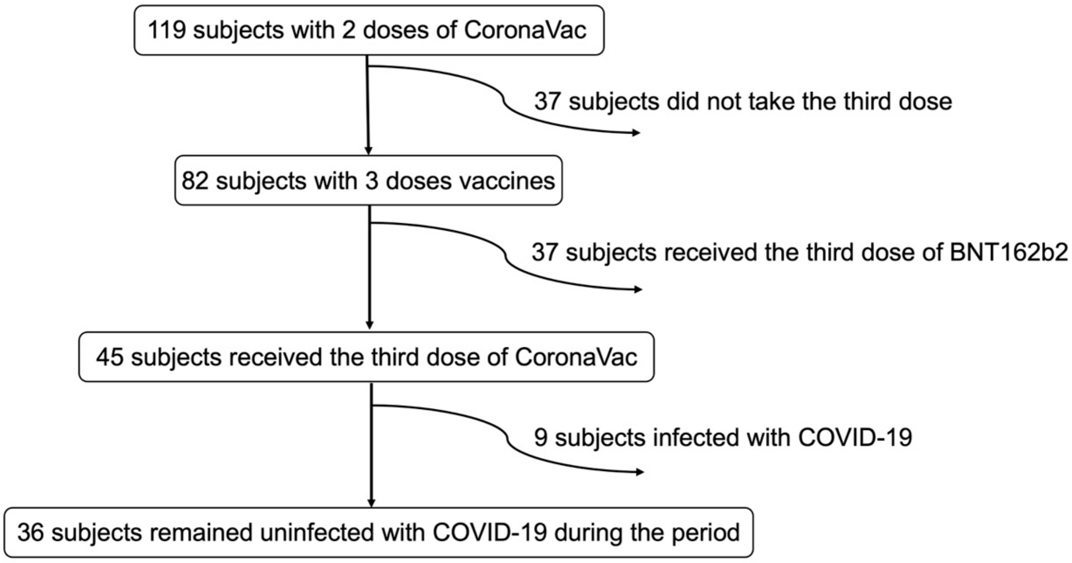

Figure S2. Receiver Operating Characteristic (ROC) curve and Area under the ROC curve (AUROC) with 95% confidence intervals for predictive models.

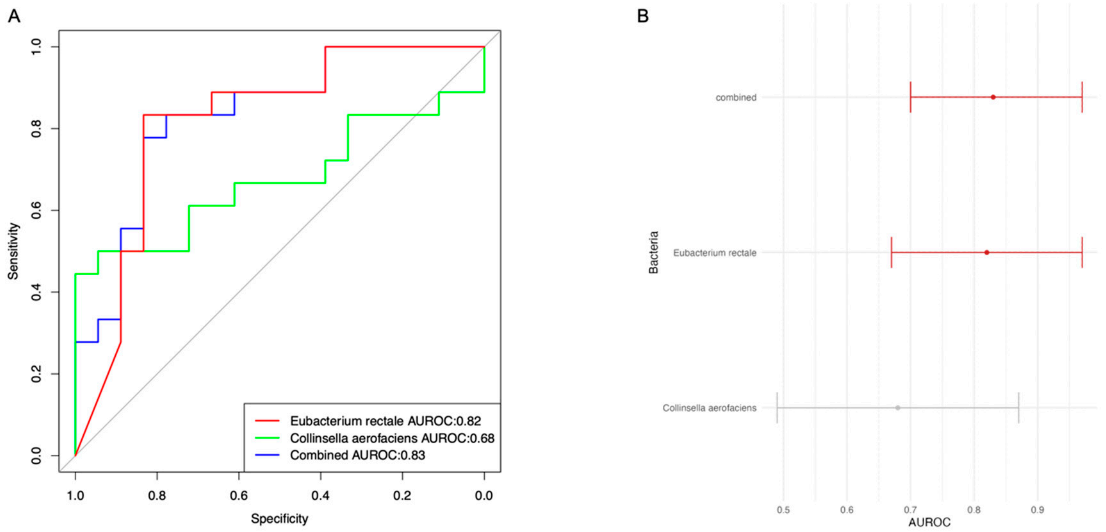

A. ROC curves of models constructed individually from species biomarkers—Eubacterium rectale, and Collinsella aerofaciens—and a combined model incorporating the two species, using generalized linear models.

B. AUROC of the predictive models. The models with AUROC greater than 0.7 were highlighted with red.

Figure S3. Differential enrichment of gut metabolic pathways in low and high immune response group based on LEfSe analysis.

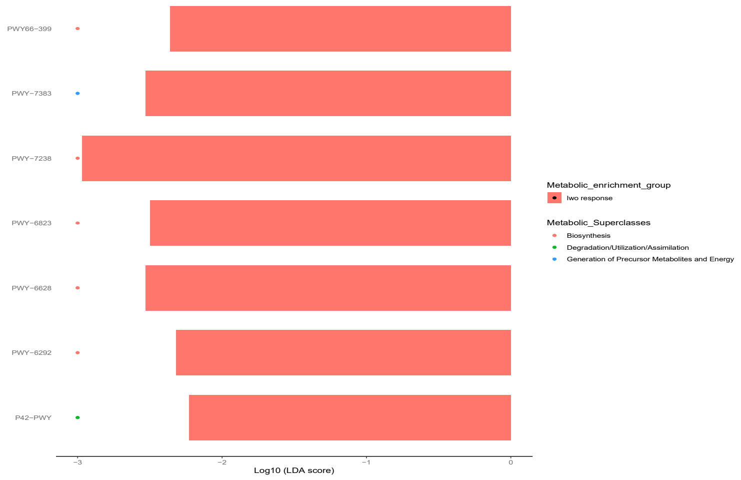

Figure S4. Correlation between metabolic pathways and virus microneutralization (vMN) titres

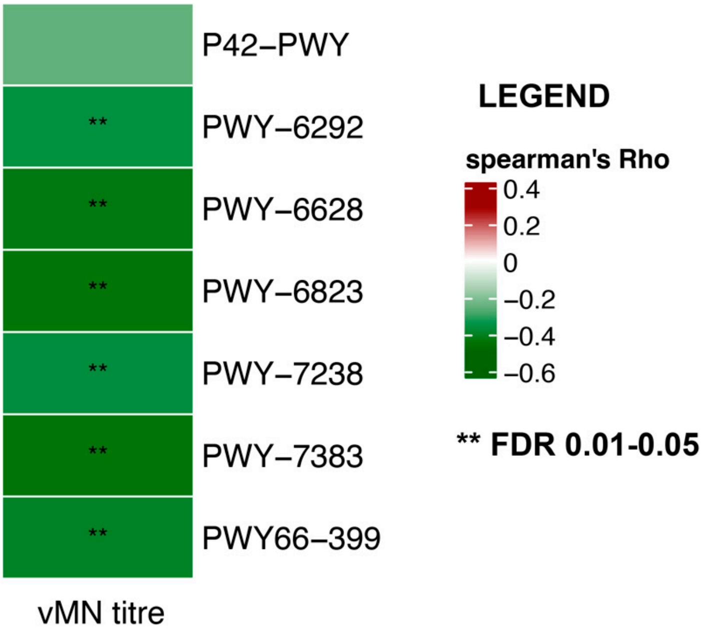

Heatmap illustrating the correlations between the relative abundance of metabolic pathways and virus microneutralization titres, analysed by Spearman's correlation analysis.

Table S1. Baseline characteristics comparison between subjects with low and high immune response at one year post-vaccination.

	Whole cohort N=36	Low reponse group N=18	High response group N=18	p-value
Age,years, (median(IQR))	52.7 (47.9-56.4)	53.35 (49.2-56.7)	52.05 (45.0-55.8)	0.602
Male (n,%)	14（38.9%）	7 (38.9%)	7 (38.9%)	1.000
BMI, kg/m2, (median (IQR))	24.2 (21.8-25.0)	24.6 (22.8-24.9)	23.5 (21.7-26.9)	0.950
OWOB (n,%)	23 (63.9%)	12 (66.7%)	11 (61.1%)	1.000
Smoking history (n,%)	3 (8.3%)	2 (11.1%)	1 (5.6%)	1.000
Drinking history (n,%)	3 (8.3%)	2 (11.1%)	1 (5.6%)	1.000
DM or pre-DM (n,%)	17 (47.2%)	7 (38.9%)	10 (55.6%)	0.505
Hypertension (n,%)	8 (22.2%)	4 (22.2%)	4 (22.2%)	1.000
MASLD (n,%)	13 (36.1%)	8 (44.4%)	5 (27.8%)	0.489
Gastrointestinal surgery (n,%)	2 (5.6%)	1 (5.6%)	1 (5.6%)	1.000
Proton pump inhibitor use* (n,%)	6 (16.7%)	4 (22.2%)	2 (11.1%)	0.658
Antibiotic use* (n,%)	2 (5.6%)	1 (5.6%)	1 (5.6%)	1.000
Fibroscore, kPa (median (IQR))	4.35 (3.28-5.78)	4.35 (3.60-5.60)	4.4 (2.98-5.90)	0.776

*usage with 1 year before first vaccination. Abbreviation: BMI, body mass index; OWOB, overweight/obese status; DM or pre-DM, diabetes mellitus or pre-diabetes mellitus; MASLD, metabolic dysfunction-associated steatotic liver disease.

Table S2. Summary of the identified metabolic pathways

Abbreviation	Full name	Superclass	Vaccinee group	LDA score	p-value
P42-PWY	incomplete reductive TCA cycle	Degradation/Utilization/Assimilation	Low response	2.23	0.018
PWY-6292	superpathway of L-cysteine biosynthesis (mammalian)	Biosynthesis	Low response	2.32	0.029
PWY-6628	superpathway of L-phenylalanine biosynthesis	Biosynthesis	Low response	2.53	0.016
PWY-6823	molybdopterin biosynthesis	Biosynthesis	Low response	2.50	0.010
PWY-7238	sucrose biosynthesis II	Biosynthesis	Low response	2.97	0.034
PWY-7383	anaerobic energy metabolism (invertebrates, cytosol)	Generation of Precursor Metabolites and Energy	Low response	2.53	0.018
PWY66-399	gluconeogenesis III	Biosynthesis	Low response	2.36	0.034

Table S3. Correlation between metabolic pathways and virus microneutralization (vMN) titres based on Spearman's correlation analysis

Pathways	Variable	Correlation coefficient	p-value	FDR
P42-PWY	vMN titre	-0.25	0.143	0.14
PWY-6292	vMN titre	-0.35	0.034	0.04
PWY-6628	vMN titre	-0.43	0.009	0.02
PWY-6823	vMN titre	-0.44	0.008	0.02
PWY-7238	vMN titre	-0.36	0.031	0.04
PWY-7383	vMN titre	-0.44	0.008	0.02
PWY66-399	vMN titre	-0.40	0.016	0.03
